# Microbiota-derived acetate attenuates neuroinflammation in rostral ventrolateral medulla of spontaneously hypertensive rats

**DOI:** 10.1186/s12974-024-03061-3

**Published:** 2024-04-18

**Authors:** Xiaopeng Yin, Changhao Duan, Lin Zhang, Yufang Zhu, Yueyao Qiu, Kaiyi Shi, Sen Wang, Xiaoguang Zhang, Huaxing Zhang, Yinchao Hao, Fang Yuan, Yanming Tian

**Affiliations:** 1 Department of Neurobiology, Hebei Medical University, Shijiazhuang, 050017 China; 2 Department of Physiology, Hebei Medical University, Shijiazhuang, 050017 China; 3 Core Facilities and Centers, Hebei Medical University, Shijiazhuang, 050017 China; 4 Hebei Province Key Laboratory of Neurophysiology, Shijiazhuang, 050017 China

**Keywords:** Hypertension, Gut microbiota, Short-chain fatty acid, Neuroinflammation

## Abstract

**Background:**

Increased neuroinflammation in brain regions regulating sympathetic nerves is associated with hypertension. Emerging evidence from both human and animal studies suggests a link between hypertension and gut microbiota, as well as microbiota-derived metabolites short-chain fatty acids (SCFAs). However, the precise mechanisms underlying this gut-brain axis remain unclear.

**Methods:**

The levels of microbiota-derived SCFAs in spontaneously hypertensive rats (SHRs) were determined by gas chromatography-mass spectrometry. To observe the effect of acetate on arterial blood pressure (ABP) in rats, sodium acetate was supplemented via drinking water for continuous 7 days. ABP was recorded by radio telemetry. The inflammatory factors, morphology of microglia and astrocytes in rostral ventrolateral medulla (RVLM) were detected. In addition, blood-brain barrier (BBB) permeability, composition and metabolomics of the gut microbiome, and intestinal pathological manifestations were also measured.

**Results:**

The serum acetate levels in SHRs are lower than in normotensive control rats. Supplementation with acetate reduces ABP, inhibits sympathetic nerve activity in SHRs. Furthermore, acetate suppresses RVLM neuroinflammation in SHRs, increases microglia and astrocyte morphologic complexity, decreases BBB permeability, modulates intestinal flora, increases fecal flora metabolites, and inhibits intestinal fibrosis.

**Conclusions:**

Microbiota-derived acetate exerts antihypertensive effects by modulating microglia and astrocytes and inhibiting neuroinflammation and sympathetic output.

**Supplementary Information:**

The online version contains supplementary material available at 10.1186/s12974-024-03061-3.

## Introduction

Hypertension is a major risk factor for cardiovascular disease, stroke, and chronic kidney disease, with a growing economic burden on the population and society [1]. The sympathetic nervous system plays a key role in the long-term regulation of arterial blood pressure by integrating neurohumoral signals and regulating cardiac, peripheral resistance vessel and renal function [2]. The onset and progression of many cardiovascular diseases, including hypertension and heart failure, are accompanied by alterations in sympathetic function [3]. The rostral ventrolateral medulla (RVLM) of the medulla oblongata is an important node in the sympathetic regulatory network, and the RVLM can project monosynaptically to spinal sympathetic preganglionic neurons, which in turn affects arterial blood pressure [4]. Factors such as respiratory-sympathetic coupling [5], oxidative stress [6], and neuroinflammation can lead to elevated excitability of pre-sympathetic neurons of the RVLM [7], causing increased sympathetic activity and hypertension.

In recent years, a large number of clinical and basic studies have shown that gut microbiota dysbiosis is closely related to the occurrence of hypertension [8–12]. Intestinal flora dysbiosis occurs in hypertension mainly manifested as a significant decrease in the abundance and diversity of the flora, and a significant decrease in the number of short-chain fatty acid-producing bacteria [11]. The fact that blood pressure was elevated in normotensive rodents that received fecal bacteria from hypertensive patients or rats; [10, 12, 13] and probiotics or dietary fibers that regulate intestinal flora have anti-hypertensive effects [14–16], suggesting that intestinal flora dysbiosis is an important pathogenesis of hypertension. However, the pathways through which microorganisms colonizing the gut affect arterial blood pressure (ABP) remain unknown.

Gut microbes interact with the brain through a variety of pathways, including the autonomic and enteric nervous systems, the immune system, and metabolites such as short-chain fatty acids (SCFAs) [17, 18]. The main source of SCFAs in the organism is the production of dietary fiber through fermentation by gut microbes. In addition to maintaining the integrity of the intestinal barrier, producing mucus, inhibiting inflammatory responses and other local effects, SCFAs are also involved in regulating the integrity of the blood-brain barrier (BBB) and the functions of microglia and astrocytes, suggesting that SCFAs play a key role in microbial-gut-brain axis communication [19–21]. SCFAs in feces and blood are highest in acetate, and SCFAs such as acetate, propionate, and butyrate can also be detected in human cerebrospinal fluid [22], but the role of SCFAs in hypertension is still controversial. Both increased and decreased plasma levels of SCFAs have been reported in hypertensive populations [23–26]. Both human and animal experiments have demonstrated that acetate can lower blood pressure in hypertension [27, 28], however, the underlying mechanism has yet to be elucidated.

## Materials and methods

### Animals and acetate administration

All treatments of animals and experimental procedures followed the guidelines of the Animal Care and Ethical Committee of Hebei Medical University (Hebmu-2,017,003). We used 12-week-old male SHRs and age-matched normotensive Wistar-Kyoto (WKY) rats purchased from the Beijing Vital River Laboratory Animal Technology Co., Ltd. (Beijing, China). Animals were housed in a clean-grade animal room with constant temperature (22 ± 1 °C) and humidity (50 ± 10%), with 12-hour day-night shift. All animals were freely access to food and water. Upon completion of the experiments, the rats were humanely euthanized with an overdose of sodium pentobarbital (> 100 mg/kg, i.p.).

To observe the effect of acetate on ABP in rats, sodium acetate (67.5 mM, S2889, Sigma-Aldrich, USA) was administered via drinking water (pH 7.4) as previously described for 7 days [29]. Drinking water was refreshed every day. The control group was given normal water without sodium acetate. ABP, body weight, diet intake, water intake and urine output were measured daily in rats.

### Quantification of SCFAs

The concentrations of SCFAs in venous blood (from inferior vena cava of rats), cerebrospinal fluid (from the fourth ventricle of rats), and feces (from colon of rats) were determined using Agilent 7890B GC-MS (Agilent Technologies, USA). Fecal samples (20 mg) were homogenized by 1 mL phosphoric acid (0.5% v/v) and 0.5 mL MTBE (containing internal standard) solution. 50 µL of plasma/cerebrospinal fluid were homogenized by 100 µL of 36% phosphoric acid solution and 150 µL of 2-methylvaleric acid internal standard solution. After centrifugation (10 min at 16,000 g and 4 °C), 90 µL of supernatant was absorbed into the sampling bottle for GC-MS/MS analysis. Data acquisition and processing was using Agilent MassHunter software. The concentration of each SCFAs was quantified by the calibration curves constructed using each SCFAs standard solution.

### ABP measurement

**Telemetry measurement**: to observe the effect of acetate on hypertension, we recorded the ABP of WKY rats and SHRs after acetate treatment for 7 days using an implanted radiotelemetry device (HD-S11, DataSciences, USA) daily (from 9:00 to 17:00). In brief, after anaesthetized with isoflurane (2.5%), blood pressure catheter of the telemeter was inserted into the abdominal aorta from the left femoral artery. The transmitter was fixed subcutaneously to the abdomen of rats. Surgery was performed under strict aseptic conditions, and rats were given penicillin (24,000 IU) for 3 days after surgery. Acetate treatment was performed 7 days after surgery. The diurnal changes in systolic blood pressure (SBP, mmHg), diastolic blood pressure (DBP, mmHg) and heart rate (HR, beats per minute, bpm) were measured after 7 days of acetate treatment over a minimum of 24 h at a sampling rate of 500 Hz (Ponemah software, DSI, USA).

**Tail-cuff measurement**: a noninvasive computerized tail-cuff blood pressure system (Kent Scientific, USA) was used to measure the ABP of 5-week-old juvenile SHRs (j-SHR) and age-matched WKY rats (j-WKY) daily (from 9:00 to 17:00). The rats were trained for continuous 7 days to accustom the tail-cuff procedure before data acquisition. SBP, DBP and HR were determined by averaging 5 effective repeats, which were verified with tracings manually reviewed.

### Evaluation of autonomic function

Autonomic function was evaluated in conscious freely moving rats after 7 days of acetate treatment using a pharmacological method. The rats were administered the β-adrenergic receptor blocker propranolol (1 mg/kg, Tocris, USA), the muscarinic cholinergic receptor blocker atropine (1 mg/kg, Tocris), and a ganglionic blocker hexamethonium (5 mg/kg, Tocris) via intraperitoneal injections. The radio telemetry system was employed to measure ABP and HR. The changes in HR (ΔHR) or mean arterial pressure (ΔMAP) were computed following the administration of these blockers. Each injection was separated by a 24-hour recovery period.

### Plasma norepinephrine measurement

Rats were anesthetized with urethane (800 mg/kg) and α-chloralose (60 mg/kg) intraperitoneally, and venous blood was drawn from the inferior vena cava. After centrifugation (2000 g, 15 min), the concentration of norepinephrine (NE) in serum was determined by an ELISA kit (ab287789, abcam, UK) according to the manufacturers’ instruction. The results were read at 450 nm using a microplate reader (PowerWaveHT, BioTek, USA).

### Immunofluorescence staining

After being anesthetized by intraperitoneal injection of urethane (1.8 g/kg), rats were perfused with chilled saline (300 ml) and phosphate-buffered paraformaldehyde solution (4%, 0.1 M, pH 7.4, 300 ml) transcardially sequentially. The rats were decapitated, and the brainstems were rapidly removed and stored in the perfusion fixative at 4 °C for 48 h, and subsequently immersed in 30% sucrose in phosphate-buffered saline (PBS) at 4 °C for at least 2 days. A series of 25 μm sections were obtained using a cryostat (CM1950; Leica Microsystems, Germany). The sections were washed by PBS for 3 times, and then immersed in blocking buffer (2% BSA in PBS) for 30 min at room temperature. For immunofluorescence staining, the tissue sections were incubated with primary antibodies at 4 °C overnight. After rinsing with PBS again, the sections were then incubated with secondary antibodies for 1 h at room temperature. The sections were examined with a laser scanning confocal microscope (FV1000, Olympus, Japan).

The following antibodies were used: mouse monoclonal anti-IBA1 (dilution 1:100, AB283319, abcam, UK); mouse monoclonal anti-tyrosine hydroxylase (dilution 1:400, MAB318, Sigma-Aldrich, USA); rabbit monoclonal anti-NeuN (dilution 1:100, # 24,307, cell signaling technology, USA); guinea pig monoclonal anti-c-Fos (dilution 1:1000, # 226,308, synaptic systems, Germany); mouse monoclonal anti-GFAP (dilution 1:800, #3670, cell signaling technology); goat anti-mouse Alexa Fluor 488 (dilution 1:1000, ab150113, abcam); goat anti-guinea pig Cy3 (dilution 1:800, # 106-165-003, Jackson Immunoresearch Laboratories Inc., USA); donkey anti-rabbit Alexa Fluor 647 (dilution 1:1000, ab150075, abcam), goat anti-mouse Alexa Fluor 555 (dilution 1:1000, ab150114, abcam).

### Cell morphological analysis

Confocal images were acquired with a 40 × objective and a z-stepsize of 1-µm (FV1000, Olympus), and only cells completely included within the borders of the image and do not overlap with one another were chosen. For each animal, 12 cells were randomly selected from the RVLM area, and the mean value for each rat was calculated. The Sholl analysis Plugin (FIJI, NIH) was used to create concentric circles with a 1 μm step size from the cell soma to determine the number of intersections at each Sholl radius. The branch lengths, number of branches, and junctions were quantified by the Analyze Skeleton Plugin. All images were taken and analyzed by three researchers who were blind to the experimental conditions.

### qPCR

Rats were anesthetized (urethane, 1.8 g/kg, i.p.) and brainstem was removed and sliced into coronal sections using a vibrotome (VT1200S; Leica Biosystems). The RVLM tissue was collected under a microscope based on the anatomical atlas. Total RNA was extracted using the RNA Extraction Kit (LS1040, Promega, China). After reverse transcription with HiScript III Super Mix, the qPCR was performed on a QuantStudio 6 real time PCR system (ABI, USA) using a qPCR kit (R323, Vazyme, China). The protocol was as follows: 95 °C for 30 s for 1 cycle; 95 °C for 10 s followed by 60 °C for 30 s for 40 cycles; 95 °C for 15 s, 60 °C for 1 min, and 95 °C for 15 s for 1 cycle. The primers were listed in Table 1. Each sample was analyzed in triplicate, and *Gapdh* was used as the internal control for normalization. The relative mRNA levels were calculated using the comparative Ct (2^−ΔΔCt^) method and normalized by WKY rats.

**Table 1:** Primers sequences

Gene	Sense/antisense	Production size (bp)
*Il1b*	(+)5′-AATCTCACAGCAGCATCTCGACAAG-3′ (-)5′-TCCACGGGCAAGACATAGGTAGC-3′	98
*Il6*	(+)5′-ACTTCCAGCCAGTTGCCTTCTTG-3′	110
	(-)5′-TGGTCTGTTGTGGGTGGTATCCTC-3′	
*Tnf*	(+)5′-ATGGGCTCCCTCTCATCAGTTCC-3′	111
	(-)5′-CCTCCGCTTGGTGGTTTGCTAC-3′	
*Gapdh*	(+)5′-CTGCACCACCAACTGCTTAG-3′	119
	(-)5′-GGCCATCCACAGTCTTCTGA-3′	

### Transmission electron microscope

After deep anesthesia with urethane (1.8 g/kg, i.p.), rats were transcardially perfused with saline and a mixture of 2% paraformaldehyde and 2.5% glutaraldehyde sequentially. The whole brain was removed, and the tissue of RVLM area was cut into small square pieces of 1 mm × 1 mm × 1 mm size according to the atlas. The tissue was fixed in 3% glutaraldehyde at 4 °C for 4 h, and then fixed in 1% osmium for 2 h. The tissue was gradient dehydrated in acetone, permeated with propylene oxide and then embedded in epoxy resin, and sliced using an ultrathin sectioning machine (Leica Microsystems) at 40 nm thickness. The sections were double-stained with uranyl acetate and lead citrate and observed under a transmission electron microscope (S7500 Hitachi, Japan).

### Evans blue leakage

After anesthetized by intraperitoneal injection of urethane (1.8 g/kg), rats were injected via femoral vein with Evans blue dye (2% concentration in saline, 3 mL/kg body weight, E8010, Solarbio, China), and underwent transcardial perfusion with saline 30 min later. Subsequently, the brains were dissected, weighed, and photographed. The dissected brains were then homogenized in a 9-fold volume of 50% trichloroacetic acid and sonicated for 2 min. After that, the samples were centrifuged at 3000 g for a duration of 20 min. The supernatants were collected and their optical density at 620 nm was measured using a spectrometer (PowerWaveHT, BioTek).

### In vivo optical imaging systems

**BBB permeability assay**: after being anesthetized by intraperitoneal injection of urethane (1.8 g/kg), rats were intravenously injected with Cy5-Dextran (10 kDa, Stargraydye, China). One hour later, after transcardial perfusion with 300 ml of PBS, the whole brain tissue was removed for observation under a 646 nm excitation wavelength exposed by a small animal live imaging system (SkyScan 1176, Bruker, Belgium) and photographed.

**Detection of supplemental acetate in the brain**: after being anesthetized by intraperitoneal injection of urethane (1.8 g/kg), rats were given an intravenous injection of Sulfo-Cyanine5-labelled acetate (Cy5-acetate, provided by Xi’an Rui Xi Biotechnology Co., Ltd., China). Thirty minutes later, the rats were transcardially perfused with 300 ml of PBS to flush out the Cy5-acetate from the blood vessels. Then the rats were exposed to a 646 nm excitation wavelength using a small animal live imaging system (SkyScan 1176, Bruker) and photographed.

### Intestinal permeability

After a 24-hour fasting period, the rats were anesthetized using isoflurane. To perform the experiment, a polyurethane cannula with an external diameter of 1.5 mm was inserted into the rectum and advanced to a position 8 cm proximal to the anal verge. FITC-Dextran (4 kDa, #68,059, Sigma-Aldrich), dissolved in saline, was then introduced into the colon at a dosage of 100 mg/kg. Following the completion of instillation, the animals were positioned head-down for 5 min to prevent any leakage of the instilled Dextran solution. After 4 h, the rats were anesthetized with urethane (1.8 g/kg, i.p.) and blood was taken from the inferior vena cava to measure the absorbance using a fluorescence microplate (SpectraMax M2, Molecular Devices, USA).

### Histology examination

Rat colon tissues were collected and immersed in a 4% paraformaldehyde solution for 48 h. Subsequently, the tissues underwent gradual dehydration using a series of ethanol gradients. Following that, the tissues were embedded in wax and sliced into sections measuring 5 μm in thickness using a microtome (Leica Microsystems). These sections were then stained with hematoxylin-eosin (HE) and Masson-trichrome, and captured using a digital camera attached to a light microscope (DM6000B, Leica Microsystems). The extent of fibrosis, thickness of tunica muscularis layer, villi length, and the number of goblet cells per villi were quantified using Image J software.

### 16S rRNA gene sequencing

After supplementing with acetate for 7 days, colonic luminal contents were collected from rats upon sacrifice. After extracting the total DNA from the samples, we assessed the DNA concentration and purity using 1% agarose gel electrophoresis. The DNA was then diluted to a concentration of 1 ng/µL using sterile water. Next, the V4 region of the bacterial 16S rDNA gene was amplified from each DNA sample using the reverse primers 515f/806r (515f: 5’-GTGCCAGCMGCCGCGGTAA-3’, 806r: 5’-GGACTACHVGGGTWTCTAAT-3’). To facilitate sequencing, a sequencing connector was added to the end of the primers, and PCR amplification was performed. The resulting products underwent purification, quantification, and homogenization to generate sequencing libraries. These libraries were then subjected to quality control assessment, and only the ones that met the quality standards were selected for sequencing using the Illumina NovaSeq PE250 sequencing platform, performed by Metware Biotechnology Co., Ltd. (Wuhan, China). High-quality reads suitable for bioinformatics analysis were carefully chosen. All valid reads from each sample were then grouped into operational taxonomic units (OTUs) using a 97% sequence similarity threshold. To evaluate the variation between the experimental groups (β-diversity), Principal Component Analysis (PCA) plots were utilized. Additionally, linear discriminant analysis coupled with effect size (LEfSe) was carried out using LEFSE software.

### Untargeted metabolomics

Metabolomics of collected fecal samples was performed by LC-MS/MS. Taken 200 mg of lyophilized feces and added to 4 mL of ultrapure water, then vortexed well for 30 s. Added 1 mL of ice-cold MeOH and H_2_O (80:20) mixture, vortexed for 1 min, centrifuged the mixture at 13,000 g for 10 min. The supernatant was collected and passed through a polyamide filter (25 mm, pore size 0.45 μm), diluted with water (1:3) and transferred to a glass vial for LC-MS/MS detection. Pooled 10 µL aliquots from each sample to create a composite quality control sample for metabolomic analysis.

An ultra-high-performance liquid chromatography system (Ultimate 3000, Thermo Fisher Scientific, USA) was employed, and the analysis was carried out with a high-resolution tandem electrostatic field Orbitrap mass spectrometer (QE Plus, Thermo Fisher Scientific). The Compound Discoverer 3.2 software facilitated the chromatographic peak identification, alignment, and normalization processes, yielding files that included m/z values, retention times (Rt), and peak areas for subsequent analysis. PCA of the collected data, differential metabolite screening and pathway enrichment were done by metaboanalyst 6.0. Differential metabolites between groups were screened using VIP > 1.0 and *p* < 0.05 as threshold.

### Statistical analysis

Statistical analysis was performed with Prism version 7 (GraphPad Software Inc., USA). Values are presented as mean ± SEM. Data were compared by Student’s t test, and one-way ANOVA or two-way ANOVA followed by Tukey’s multiple comparisons test. Differences between groups with *P* < 0.05 were considered significant.

## Results

### The reduced serum acetate levels in SHRs

**Fig. 1:**
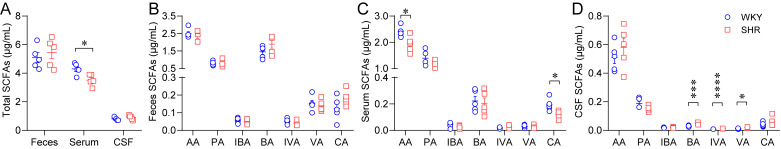
Levels of short-chain fatty acids (SCFAs) in spontaneously hypertensive rats (SHRs). A, total levels of SCFAs in feces, serum, and cerebrospinal fluid (CSF) in Wistar-Kyoto (WKY) rats and SHRs. B-D, fecal, serum and cerebrospinal fluid levels of each SCFAs in WKY rats and SHRs. *n* = 5 for each phenotype. AA, acetic acid; PA, propionic acid; IBA, isobutyric acid; BA, butyric acid; IVA, isovaleric acid; VA, valeric acid; CA, caproic acid. * *P* < 0.05, *** *P* < 0.001, **** *P* < 0.0001

To explore the potential role of SCFAs in the development of hypertension, SCFAs levels in the feces, blood and cerebrospinal fluid of WKY rats and SHRs were analyzed. We found no statistically significant difference in stool SCFAs between WKY rats and SHRs (Fig. 1A, B). Serum total SCFAs were significantly lower in SHRs than in WKY rats (Fig. 1A), with acetic acid (Fig. 1C) and caproic acid (Fig. 1C) being significantly lower. Concentrations of butyric acid, isovaleric acid and valeric acid in cerebrospinal fluid were significantly higher in SHRs than in WKY rats (Fig. 1D), whereas total SCFAs did not differ between the two groups of rats (Fig. 1A).

### Acetate decreased ABP in SHRs

**Fig. 2:**
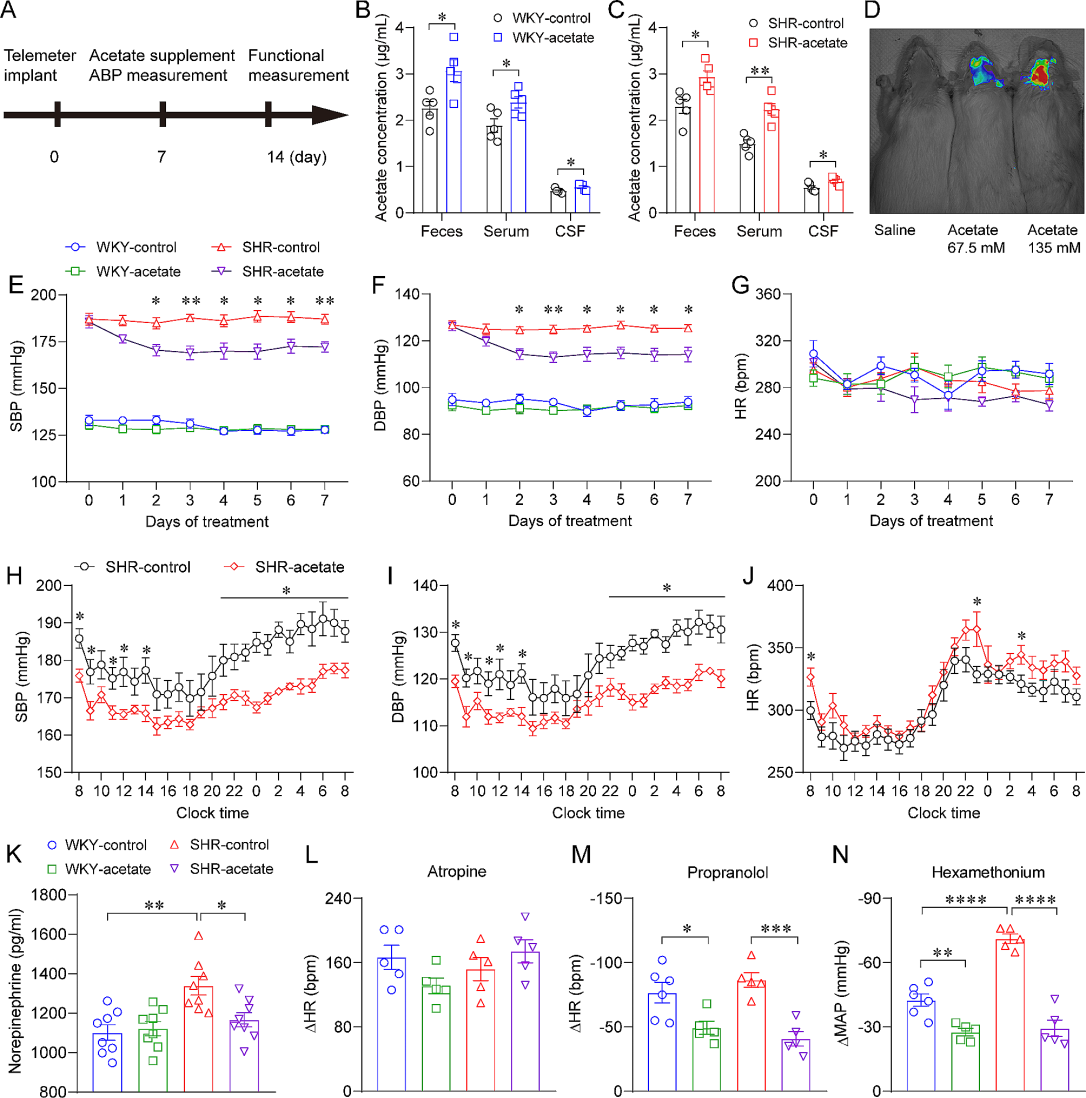
Acetate reduces arterial blood pressure (ABP) in SHRs. A, schematic diagram for implant operation and acetate treatment. B-C, increased acetate levels in feces, serum and cerebrospinal fluid of WKY rats and SHRs after 1 week of acetate treatment. *n* = 5 for each group. D, fluorescent signals detected in brain tissues after intravenous injection of Cy5-labeled acetate in WKY rats. E-G, acetate treatment reduces SBP and diastolic blood pressure (DBP) without altering heart rate (HR) in SHRs. *n* = 7 for each group. H-J, effects of acetate treatment for 1 week on 24-h ambulatory blood pressure in SHR rats. *n* = 10 for each group. K, 1-week acetate treatment reduced serum norepinephrine levels in SHRs. *n* = 8 for each group. L, tachycardic response induced by intraperitoneal injection of atropine; M, propranolol-induced bradycardia response; N, hypotensive effect induced by hexamethonium. *n* = 5 for each group. * *P* < 0.05, ** *P* < 0.01, *** *P* < 0.001, **** *P* < 0.0001

To observe the effect of acetate on ABP in SHRs, rats were given acetate in their drinking water and blood pressure was measured using the radiotelemetry daily (Fig. 2A). Acetate concentrations in feces, blood, and cerebrospinal fluid were elevated by approximately 25% after 7 days of acetate treatment in both WKY rats and SHRs (Fig. 2B, C). However, the other types of SCFAs were not affected (Figure S1). To clarify whether circulating acetate can cross the BBB, we injected Cy5-labeled acetate intravenously. After perfusion, fluorescent signals were observed in brain tissue only (Fig. 2D), and the results suggested that acetate can enter brain tissue through the BBB. It has been shown that acetate promotes obesity in rodents by increasing their food intake [30]. We therefore observed the effect of acetate treatment on the intake and body weight of rats. We found acetate had no significant effect on body weight (Figure S2A) or dietary intake (Figure S2B), but significantly increased water intake (Figure S2C) and urine output (Figure S2D), with no significant difference between the two strains of rats.

We found acetate treatment decreased SBP (Fig. 2E) and DBP (Fig. 2F) in SHRs but did not alter blood pressure in WKY rats. In addition, acetate had no significant effect on HR in both groups of rats (Fig. 2G). Moreover, we performed telemetric monitoring of 24-hour ambulatory ABP in SHRs after 7 days of acetate administration. Consistently, acetate decreased the 24-hour SBP (Fig. 2H) and DBP (Fig. 2I) in SHRs, with no significant effect on HR (Fig. 2J). To clarify whether the antihypertensive effect of acetate is associated with altered autonomic function, we examined plasma levels of NE, and analyzed the response of HR or MAP to partial blockade of the autonomic functions using pharmacological methods. Plasma NE levels were elevated in SHRs compared to WKY rats, indicating sympathetic overactivation (Fig. 2K). Plasma NE concentrations were significantly lower in the acetate-treated SHRs than in the control SHRs (Fig. 2K). Furthermore, robust elevation of HR was observed upon intraperitoneal administration of atropine, with no significant variation observed among the four groups (Fig. 2L). Intraperitoneal administration of propranolol resulted in a reduction of HR, with a lower degree of effect observed in the acetate-treated group compared to the respective control group in both genotypes (Fig. 2M). In the presence of hexamethonium, a more significant decrease in blood pressure was observed in SHRs compared to WKY rats (Fig. 2N). Additionally, within both genotypes, the acetate-treated group displayed a smaller decrease in magnitude when compared to their respective control group (Fig. 2N). Collectively, these findings indicate an acetate-dependent sympathovagal regulation characterized by decreased sympathetic drive and unaltered parasympathetic activity in SHRs.

### Acetate inhibits neuroinflammation in RVLM in SHRs

**Fig. 3:**
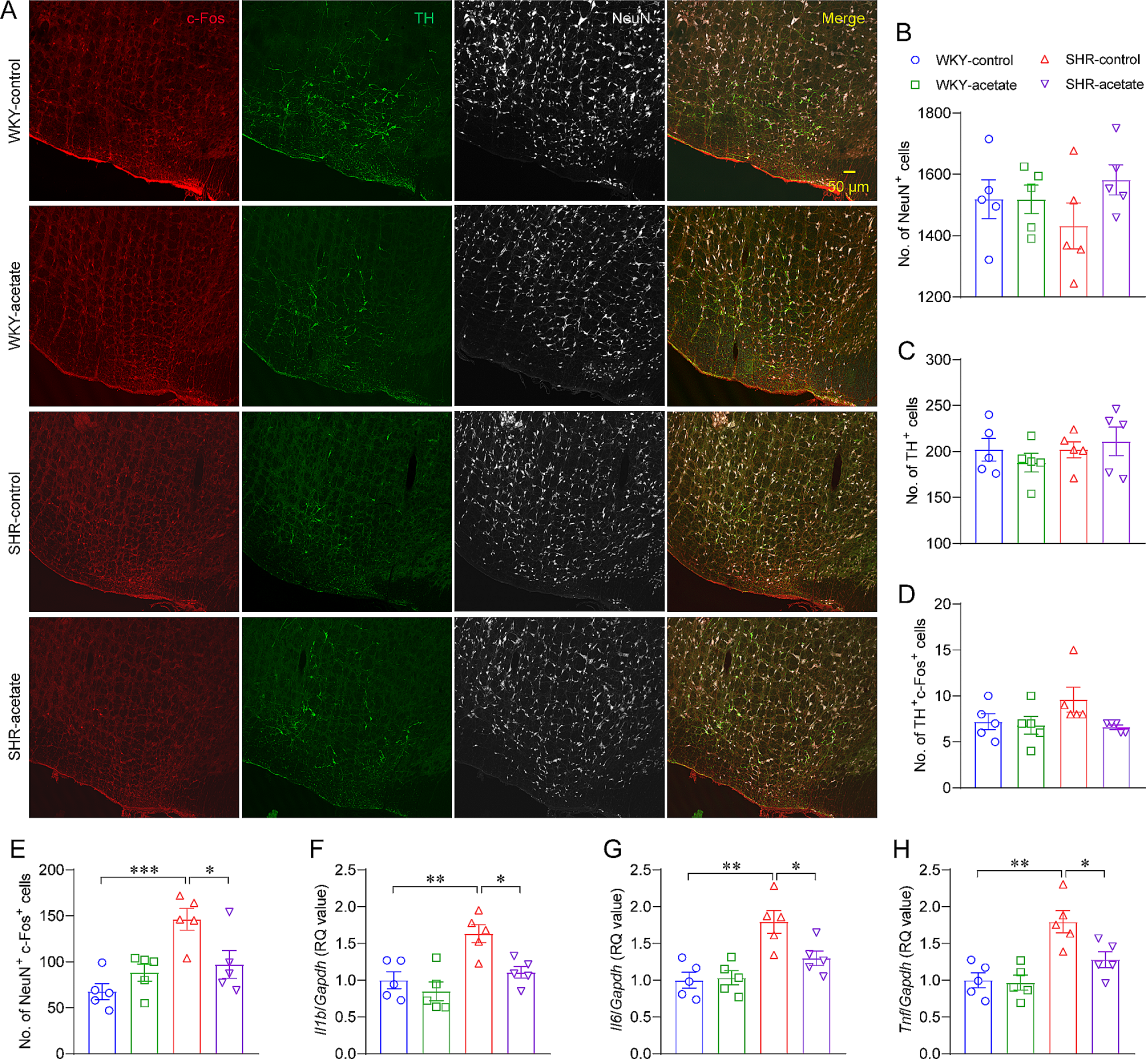
Acetate inhibited neuroinflammation in rostral ventrolateral medulla (RVLM) in SHRs. A, immunofluorescence photomicrographs showing triple labelling of c-Fos (red), tyrosine hydroxylase (TH, green) and neuronal nuclei (NeuN, white) in RVLM (bregma: −12.3 mm) of each group. B-E, the number of NeuN^+^, TH^+^, TH^+^c-Fos^+^ and NeuN^+^c-Fos^+^ neurons in each group. F-H, relative expression of inflammatory factors such as interleukin 1 beta (*Il1b*), interleukin 6 (*Il6*) and tumor necrosis factor (*Tnf*) mRNA in the RVLM of rats in various groups by qPCR. *n* = 5 for each group. * *P* < 0.05, ** *P* < 0.01, *** *P* < 0.001

To clarify whether the acetate-induced inhibition of sympathetic activity in SHRs is related to the RVLM, a crucial brainstem nucleus involved in regulating sympathetic activity, we quantified the number of activated RVLM neurons labeled with c-Fos by immunofluorescence staining (Fig. 3A). There were no differences in the number of NeuN^+^, TH^+^, or TH^+^c-Fos^+^ neurons among the four groups of rats (Fig. 3B–D). The number of NeuN^+^c-Fos^+^ neurons was significantly increased in SHRs compared to WKY rats and significantly decreased after acetate treatment (Fig. 3E). Furthermore, we employed qPCR to assess the expression levels of inflammatory factors in RVLM tissues to elucidate the potential association between alterations in RVLM neuronal excitability and neuroinflammation. It was found that *Il1b*, *Il6* and *Tnf* mRNA levels were up-regulated in SHRs compared to WKY rats (Fig. 3F–H), and down-regulated in acetate-treated SHRs compared to control SHRs (Fig. 3F–H). These findings suggest that acetate can effectively inhibit neuroinflammation and attenuate the hyperactivation of neurons in the RVLM in SHRs.

### Acetate regulates microglia morphological changes in SHRs

**Fig. 4:**
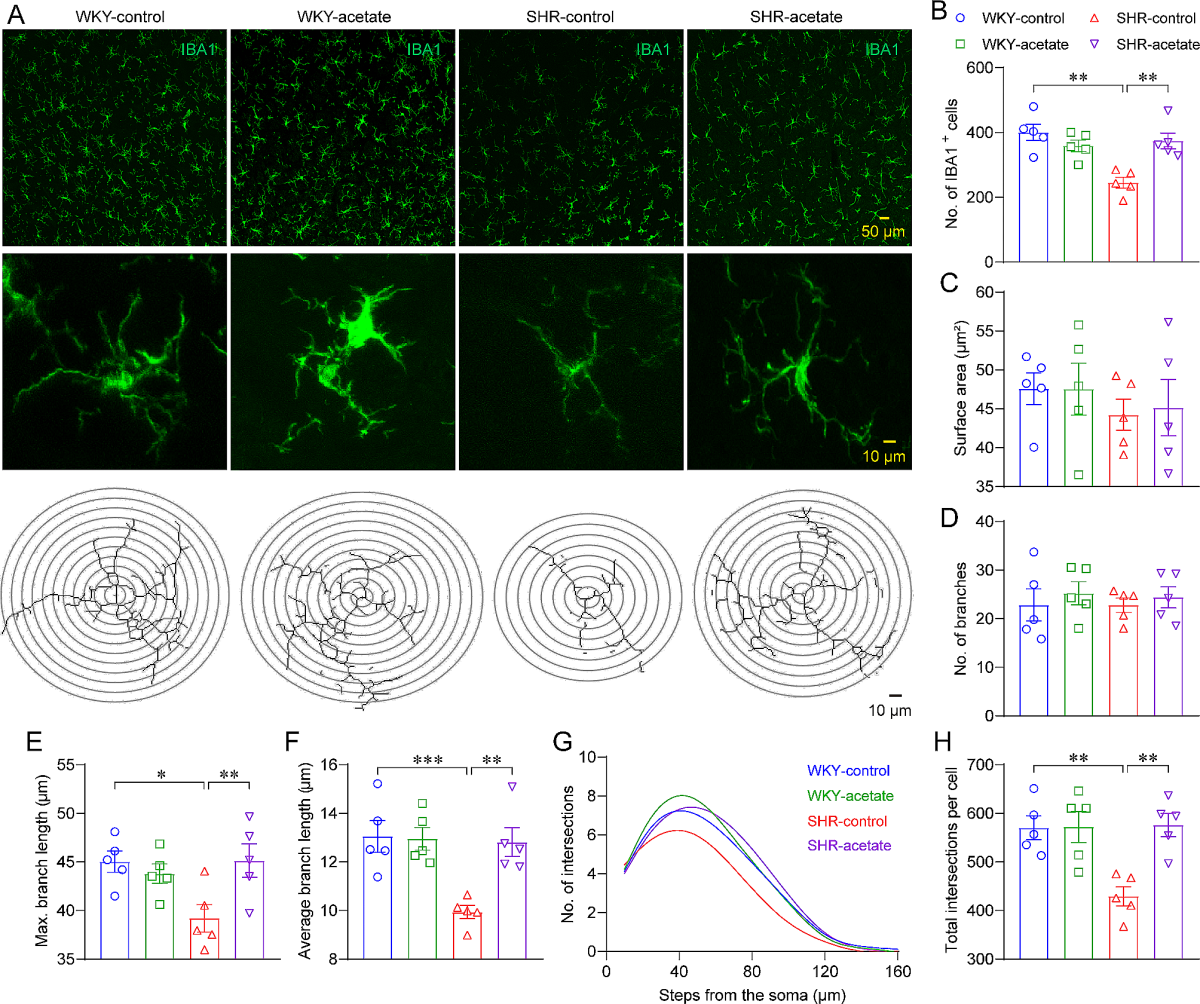
Acetate regulation of microglia morphology in the RVLM of SHRs. A, illustrative figures showcasing the expression of ionized calcium-binding adapter molecule 1 (IBA1) in the RVLM (top panes), along with photomicrographs of distinct IBA1-positive microglia (middle panes), accompanied by their respective traced contours in the lower panels (bottom panes). B-F, quantification of the density, soma size, number of branches, the maximal and average branches length of IBA1^+^ microglia. G, a non-linear curve fitting represents the average count of microglial branch intersections per 10 μm increment away from the cell body, as determined through Sholl analysis. H, the total area under the curve (AUC) of individual microglia, derived from Sholl analysis. *n* = 5 for each group. * *P* < 0.05, ** *P* < 0.01, *** *P* < 0.001

Microglia and astrocytes in the central nervous system are potential mediators in neuroinflammation [31]. To assess the involvement of microglia in neuroinflammation, we quantified numbers and morphological complexity of microglia in RVLM using confocal microscopy on IBA1^+^ stained sections respectively (Fig. 4A). In RVLM, the densities of IBA1^+^ microglia were lower in SHRs compared to WKY rats (Fig. 4B), while in acetate-treated SHRs, they were higher compared to control SHRs (Fig. 4B). There were no significant differences in the soma size (Fig. 4C) or numbers of branches (Fig. 4D) among the four groups of rats. Further analysis of microglial morphology indicated that SHRs had shorter branch reaches (Fig. 4E) and decreased average branch length (Fig. 4F) compared to WKY rats. Moreover, we found that alterations in microglia morphology in SHRs occurred as early as 5 weeks of age before the onset of hypertension (Figure S3-4). However, acetate-treated SHRs displayed longer maximum branch length and average branch length compared to control SHRs (Fig. 4E, F). The morphological analysis of microglia indicated a more branched morphology in WKY rats compared to SHRs (Fig. 4G), as shown by the area under the curve (AUC) of the Sholl analysis (Fig. 4H). Additionally, the SHRs treated with acetate exhibited an even more elaborate microglia morphology compared to the control SHRs (Fig. 4G, H).

### Acetate regulates astrocyte morphological performance in SHRs

**Fig. 5:**
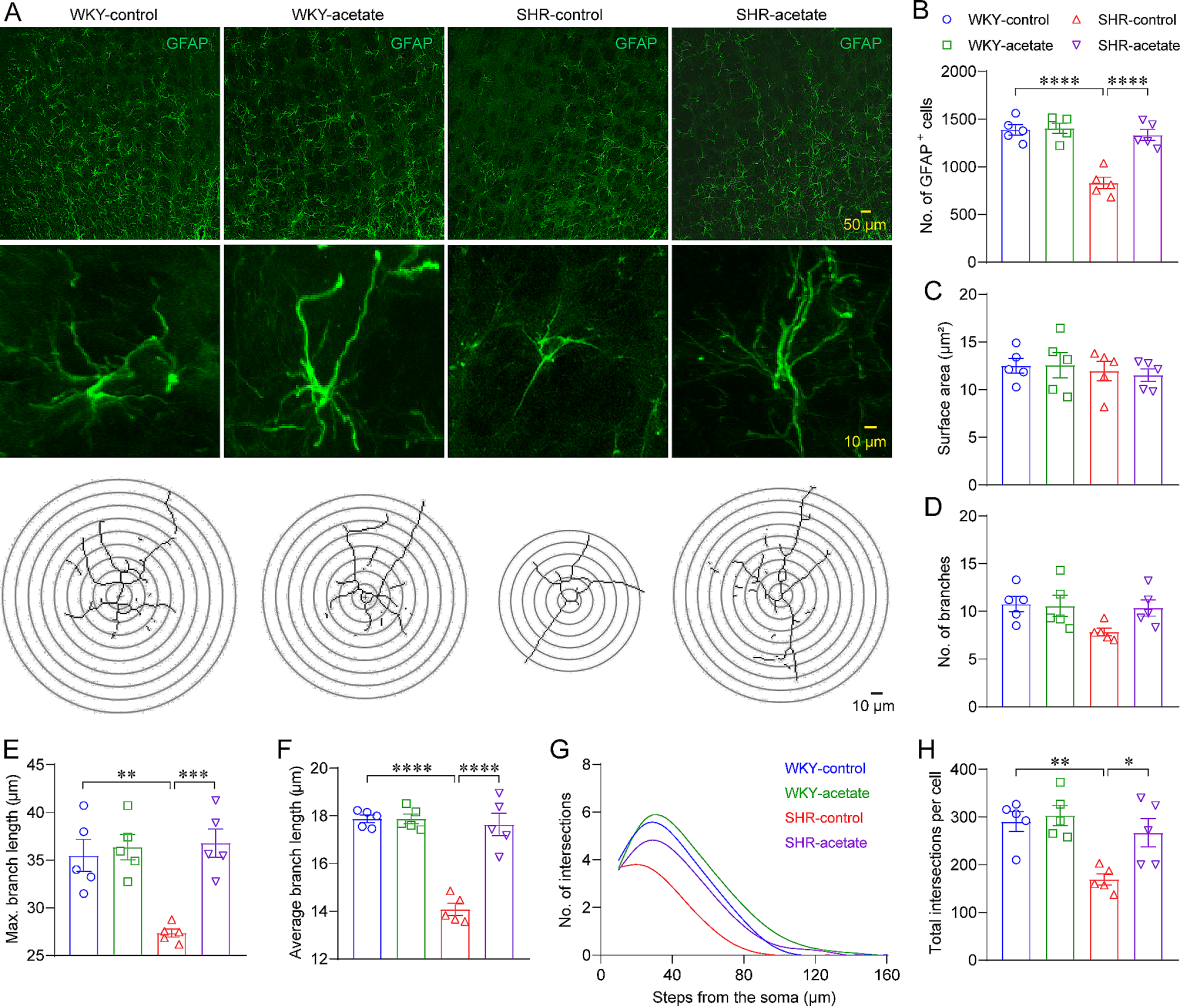
Effect of acetate on the morphology of astrocytes in the RVLM of SHRs. A, representative images displaying glial fibrillary acidic protein (GFAP) expression in the RVLM were presented in the top panels, the middle panels feature micrographs of individual GFAP-positive astrocytes, while the traced outlines of these cells were displayed in the bottom panels. B-F, assessment of GFAP-positive astrocytes characteristics such as cell density, size of soma, count of branches, and both the longest and average lengths of the branches. G, a non-linear curve fitting illustrates the average frequency of branch intersections per 10 μm from the astrocytes cell soma, as calculated using Sholl analysis. H, the AUC ascertained from Sholl analysis for individual astrocyte. *n* = 5 for each group. * *P* < 0.05, ** *P* < 0.01, *** *P* < 0.001, **** *P* < 0.0001

Because astrocytes are influenced by microglia in neuroinflammation [32], we analyzed the number and morphology of astrocytes (Fig. 5A). Firstly, the number and morphology of astrocytes in juvenile SHR were not significantly different from those of age-matched WKY rats (Figure S5). In adult rats, we found that the density (Fig. 5B) and morphological complexity (Fig. 5G, H) of astrocytes were reduced in SHRs compared to WKY rats, and which was significantly increased in acetate treated SHRs compared to control SHRs. More specifically, it was observed that SHRs exhibited shorter longest branch lengths (Fig. 5E) and shorter average branch lengths (Fig. 5F) compared to WKY rats. However, in SHRs treated with acetate, there was a significant increase in longest branch lengths and average branch lengths compared to the control SHR group (Fig. 5E, F). No differences were found in soma size or branches number among the four groups of rats (Fig. 5C, D). The observation revealed a decreased morphological complexity of microglia and astrocytes in the RVLM of SHRs compared to WKY rats. However, acetate treatment resulted in an increase in microglia and astrocytes morphological complexity in SHRs.

### Acetate improves BBB integrity in SHRs

**Fig. 6:**
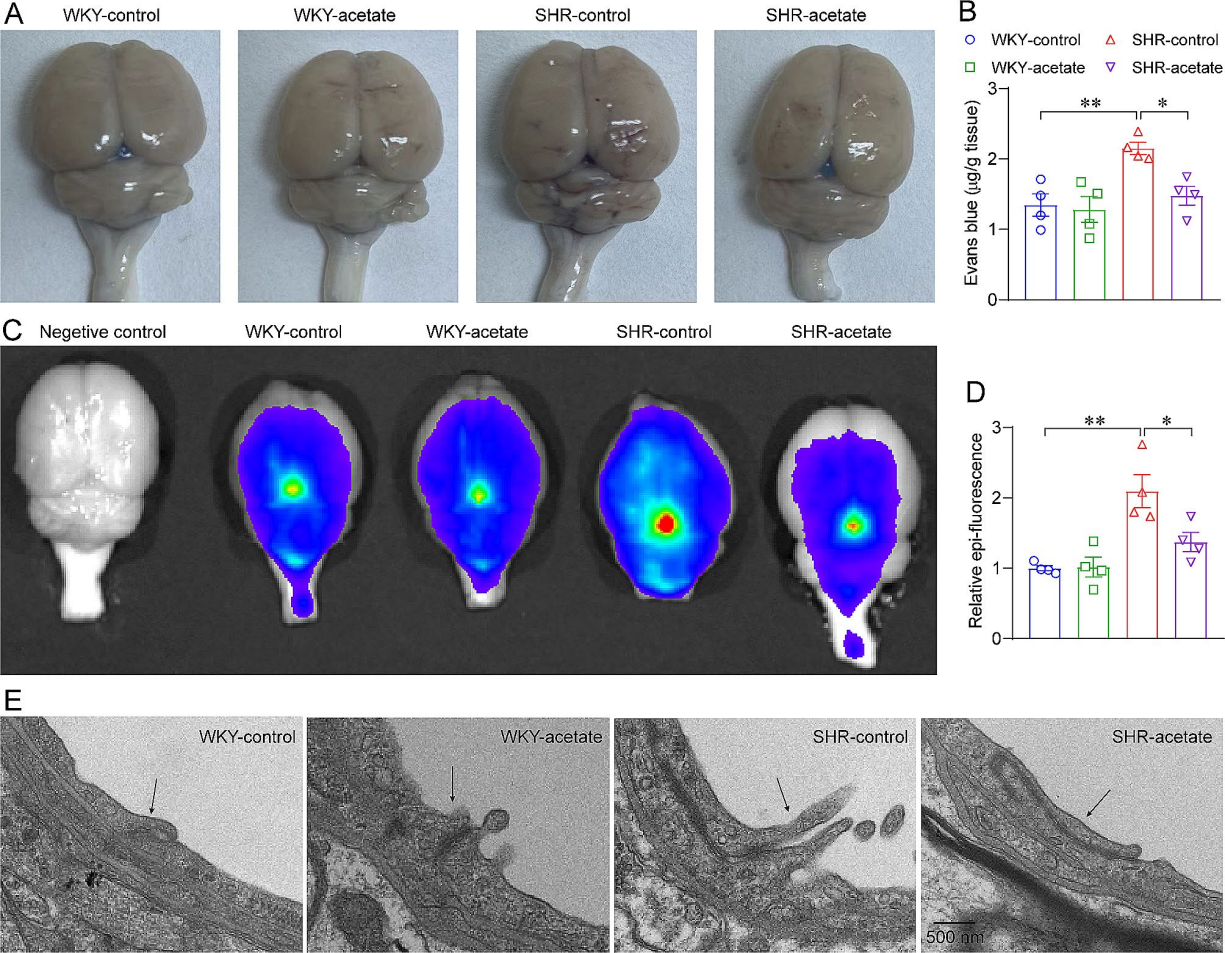
Acetate reduces blood-brain barrier (BBB) permeability in SHRs. A, assessment of BBB permeability by injection of Evans blue dye. B, OD_620_ readings of the brain tissues following Evans blue dye injection. C, application of small animal imaging to assess BBB permeability by injection of Cy5-Dextran (10 kDa). D, the relative Epi-fluorescence of brains in each group rats. *n* = 4 for each group. * *P* < 0.05, ** *P* < 0.01.E, transmission electron microscopic observation of vascular endothelial tight junction structure in the medulla of rats. The tight junctions between capillary endothelial cells are indicated by the arrows

Disruption of the BBB is frequently observed in neuroinflammatory neurological disorders [33]. Astrocytes are involved in the composition of the BBB, and microglia regulate the integrity of the BBB [34]. To observe the effect of acetate on BBB permeability in SHRs, Evans blue dye was intravenously injected into rats. Evans blue leakage was obviously observed in the brain tissues of SHRs (Fig. 6A), indicating an increased BBB permeability in hypertensive rats. Quantitative data showed that the brains of the SHRs displayed an approximately 60% increase in the OD_620_ value than WKY rats (Fig. 6B), which was decreased after acetate treatment (Fig. 6B). To further confirm the increased BBB permeability in SHRs, we intravenously injected the rats with the BBB-impermeable fluorescent Cy5-Dextran tracer (10-kDa), and examined by in vivo imaging technique (Fig. 6C). Similarly, SHRs have an increased fluorescence intensity in brain than WKY rats (Fig. 6D). And the fluorescence intensity in acetate treated SHRs was significantly decreased compared to control SHRs (Fig. 6D). Morphologically, we utilized transmission electron microscopy to investigate the ultrastructure of tight junctions (Fig. 6E). Our findings revealed that WKY rats exhibited intact tight junctions, while SHRs had widened gaps at the tight junctions. Additionally, acetate-treated SHRs exhibited narrowed gaps at the tight junctions in comparison to the control SHRs. The results suggest that acetate treatment partially ameliorated the elevated BBB permeability in SHRs.

### Acetate improves gut dysbiosis in SHRs

**Fig. 7:**
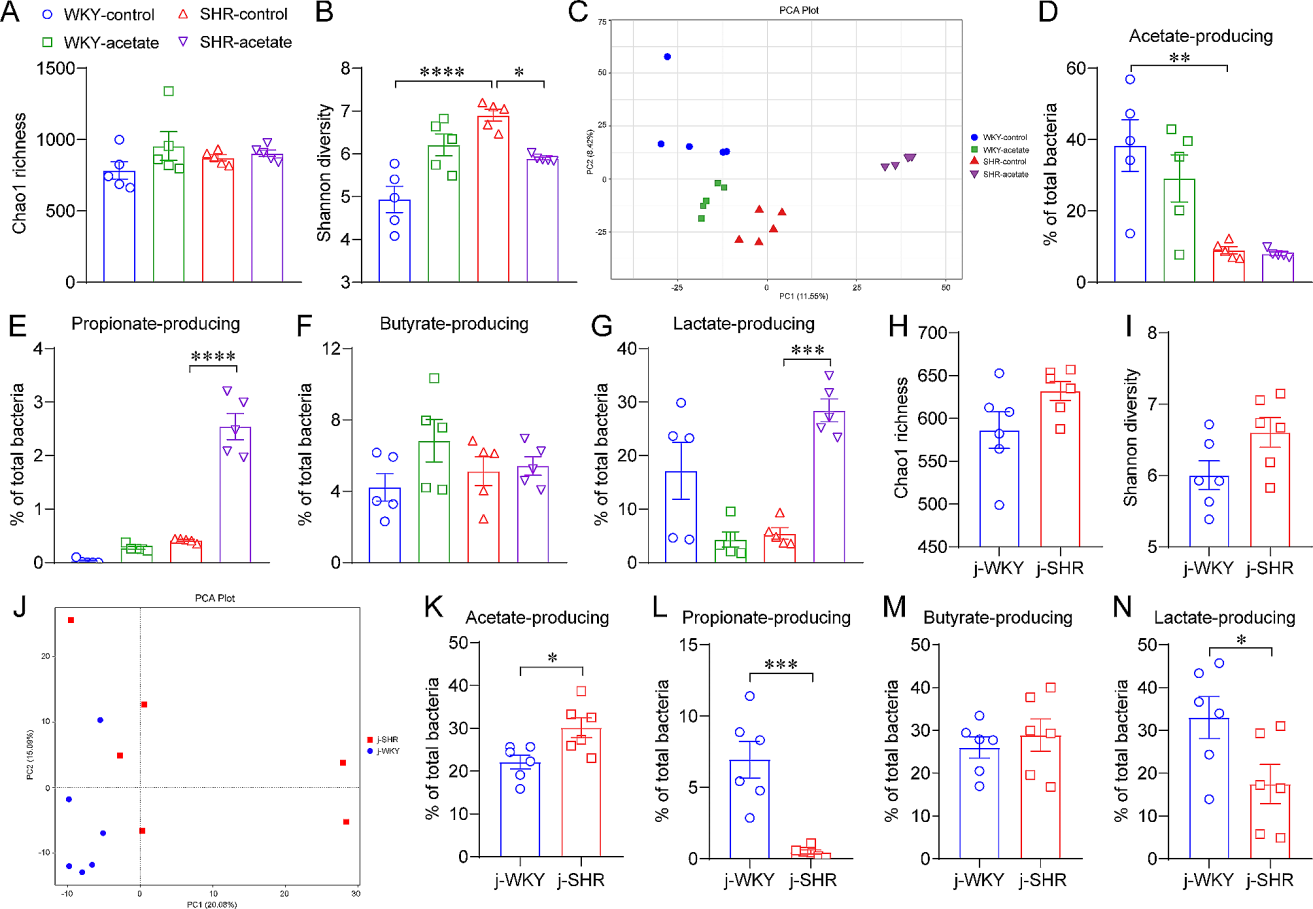
Acetate modulation of the gut microbiota in SHRs. A-B, α-diversity of gut microbiota detected by 16 S rRNA sequencing. C, the principal component analysis (PCA) based β-diversity depicts the clustering of gut microbial communities across various groups. D-G, effect of acetate supplementation on the relative abundance of acetate-, propionate-, butyrate- and lactate-producing bacteria in rats. *n* = 5 for each group. H-I, α-diversity of gut microbiota in 5-week-old juvenile SHRs (j-SHRs) and age-matched WKY rats (j-WKYs). J, PCA analysis of β-diversity in the intestinal flora of juvenile rats. K-N, relative abundance of acetate-, propionate-, butyrate- and lactate-producing bacteria in juvenile rats. *n* = 6 for each group. * *P* < 0.05, ** *P* < 0.01, *** *P* < 0.001, **** *P* < 0.0001

We conducted 16 S rRNA sequencing to examine the effects of acetate on the changes of gut microbiota in SHRs, given that it contributes to the development of hypertension. There was no significant difference in Chao1 richness between groups (Fig. 7A). The Shannon diversity showed a significant increase in SHRs compared to WKY rats (Fig. 7B). However, acetate treatment restored the changes in SHRs (Fig. 7B), suggests that acetate regulates α-diversity of the intestinal microbiota in SHRs. In addition, the PCA revealed a distinct clustering of the microbiota composition, as evidenced by the β-diversity (Fig. 7C). We then identified the relative abundance of bacteria at the phyla and genera levels (Figure S6). To clarify whether the reduced serum acetate of SHRs is related to the intestinal flora, we analyzed the abundance of acetate-producing bacteria according to the literature [35]. There was a significant decrease in acetate-producing bacterial communities in SHRs compared to WKY rats (Fig. 7D). While propionate-, butyrate- and lactate-producing bacteria levels remained stable in SHRs (Fig. 7E–G). Additionally, the administration of acetate significantly amplified the population of propionate- and lactate-producing bacteria in SHRs (Fig. 7E and G).

To determine whether the reduction of acetate-producing bacteria of SHRs was a secondary response to high ABP, we examined the gut microbiota of 5-week-old juvenile SHRs and age-matched WKYs. No difference was found in Chao1 richness or Shannon diversity between the two groups (Fig. 7H and I), suggesting that α-diversity was not significantly different between the two groups. The β-diversity was comparative analysis by PCA (Fig. 7J), and the relative abundance of bacteria at the phyla and genera levels was identified (Figure S7). Compared to juvenile WKYs, the relative abundances of acetate-producing bacteria were increased (Fig. 7K), propionate- and lactate-producing bacteria were decreased (Fig. 7L and N), butyrate-producing bacteria levels was unchanged in juvenile SHRs (Fig. 7M). These results suggest that SCFAs-producing bacteria in the gut microbes of juvenile and adult SHRs are differentiated.

### Effects of acetate on fecal metabolomics in SHRs

**Fig. 8:**
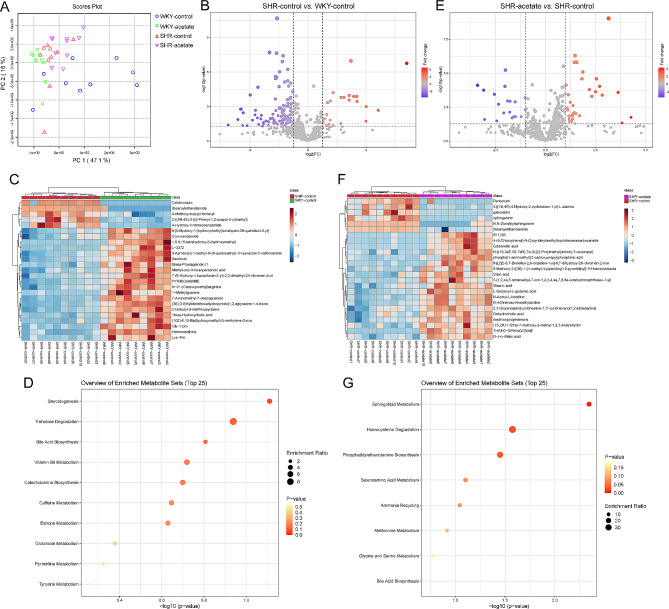
Acetate significantly increased intestinal flora metabolites in SHRs. A, PCA analysis showing spatial division for the fecal metabolome. B, the volcano plot graph of altered metabolites in control WKY rats and SHRs. C, the heatmap displays the top 25 metabolites with significant differential abundance between control WKY rats and SHRs. D, bubble plot showing the differential metabolic pathways analysis of fecal metabolites in control WKY rats and SHRs. E, the volcano plot illustrates the profile of metabolites altered in SHRs treated with acetate compared to control SHRs. F, the heatmap depicts the 25 most significantly differentially abundant metabolites when comparing acetate treatment SHRs to control SHRs. G, the bubble plot presents an analysis of the differential metabolic pathways associated with fecal metabolites in SHRs treated with acetate versus control SHRs

To explore the association between the impact of acetate on gut microbiota and alterations in the metabolome, we carried out an untargeted metabolomic analysis of fecal specimens. PCA revealed that the fecal metabolomes differed significantly across the four rat cohorts (Fig. 8A). Among the 827 metabolites identified, 147 exhibited differences between SHR and WKY rat strains, with 28 metabolites upregulated in SHRs and 119 downregulated (Fig. 8B, C). Pathway analysis (referenced to the small molecule pathway database, SMPDB) indicated an upregulation of the estrone metabolism pathway and downregulation of the steroidogenesis, trehalose degradation, bile acid biosynthesis, vitamin B6 metabolism, catecholamine biosynthesis, caffeine metabolism, glutamate metabolism, pyrimidine metabolism and tyrosine metabolism pathways in SHRs compared to WKY rats (Fig. 8D). Among the metabolites resolved in acetate-treated SHRs, 54 showed differences compared to control SHRs, with 35 being up-regulated and 19 down-regulated (Fig. 8E, F). Pathway enrichment analysis of these differential metabolites primarily revealed an up-regulation of the sphingolipid metabolism, homocysteine degradation, phosphatidylethanolamine biosynthesis, selenoamino acid metabolism, ammonia recycling, methionine metabolism, glycine and serine metabolism and bile acid biosynthesis pathways in acetate-treated SHRs compared to control SHRs (Fig. 8G). The findings suggest that acetate treatment may substantially enhance the abundance of gut microbiota-derived metabolites in SHRs.

### Acetate prevented colonic pathology in SHRs

**Fig. 9:**
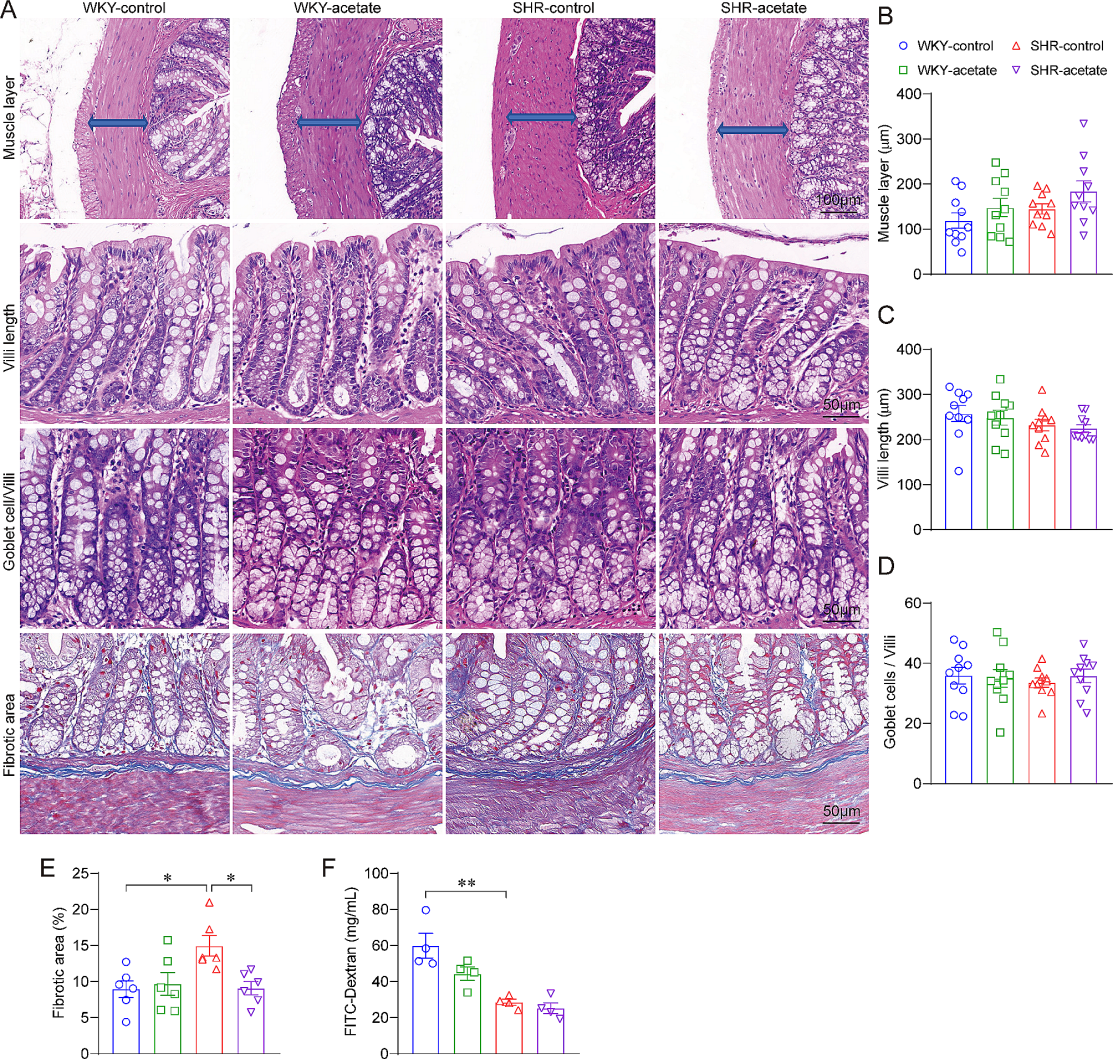
Acetate inhibits colonic tissue fibrosis in SHRs. A, representative micrographs from hematoxylin-eosin (HE) and Masson’s trichrome staining assays of the colonic tissues. Quantitative analysis of the thickness of the muscular layer (B), villi length (C) and goblet cells/villi (D) on HE-stained sections of the colon. *n* = 10 for each group. E, quantitative analysis of fibrotic area on Masson’s-stained sections of the colon. *n* = 6 for each group. F, the permeability of the colonic mucosa to the bloodstream was evaluated by measuring the plasma concentration of FITC-dextran (4 kDa). *n* = 4 for each group. * *P* < 0.05, ** *P* < 0.01

Research has demonstrated a link between intestinal pathology and hypertension [36]. Therefore, we investigated the impact of acetate supplement on both the pathological conditions and the permeability of the colon in SHRs (Fig. 9A). There were no significant differences in the thickness of the colonic smooth muscle layer (Fig. 9B), the villi length (Fig. 9C), or the number of goblet cells/villi (Fig. 9D) between WKY rats and SHRs. Furthermore, these parameters remained unchanged after acetate treatment. The fibrotic area was significantly larger in SHRs compared to WKY rats (Fig. 9E), and it showed a significant reduction following treatment with acetate (Fig. 9E). Colonic permeability was markedly reduced in SHRs in comparison to WKY rats (Fig. 9F); however, acetate treatment did not produce a significant impact on colonic permeability in either group.

## Discussion

In the present study, we demonstrated serum SCFAs and acetate were significantly lower in SHRs than in normotensive control rats. Supplementation of acetate with drinking water lowered ABP in SHRs, inhibited neuroinflammation in the RVLM, modulated microglia and astrocyte morphology, decreased BBB permeability, improved gut microbiota dysbiosis, regulated the metabolites of intestinal flora and reduced intestinal fibrosis.

Imbalances in the gut microbiota are intricately linked to the emergence and progression of hypertension [37], yet the exact mechanisms remain elusive. Numerous clinical studies in recent years have consistently shown that the microbial composition of hypertensive individuals deviates markedly from that of normotensive subjects [38–40]. Additionally, evidence from fecal transplant experiments, where flora from hypertensive donors induced blood pressure rises in mice [10], hints at a potential connection, but a definitive causal link between gut microbiota imbalances and hypertension has yet to be established. In various animal models, hypertension that arose through disparate mechanisms was each accompanied by gut microbial imbalances [11–13, 41], hinting that such dysbiosis might either be a universal pathway to hypertension or a subsequent effect of the condition. Hypertension is known to decrease intestinal blood flow [36], disrupt immune function [42], and trigger structural and functional changes within the gut [43], all of which can negatively impact the microbial landscape. And by fostering detrimental bacterial growth or diminishing beneficial species, which is a caveat noted in studies involving fecal transplants. Our current research observed notable differences in the gut microbiota composition of juvenile SHRs (prior to the development of high blood pressure) compared to age-matched normotensive WKY rats. Intriguingly, these differences were distinct from those observed in adulthood between the two groups. These findings suggest that alterations in gut microbiota could be a driving force in the onset or advancement of hypertension and that the condition of hypertension may further aggravate gut microbiota imbalances.

SCFAs are significant metabolites produced by gut microbiota, predominantly derived from the fermentation of dietary fibers. Notably, SCFA concentrations are substantially reduced in germ-free mice [44], indicating a reliance on gut flora for production. While hypertensive individuals exhibit an altered gut microbial composition, existing literature presents varied findings regarding SCFA levels in such patients [23–26]. This inconsistency may partly stem from the fact that SCFAs are highly dependent on dietary intake. Additionally, SCFA absorption through epithelial cell transport, together with fecal and urinary excretion, and the presence of SCFA-producing gut bacteria, influence SCFA levels in the bloodstream. In present study, we found significantly reduced serum acetate levels in SHRs compared to WKY rats. This finding is consistent with lower levels of acetate-producing bacteria in the intestines of SHRs, as observed in both our current and previous studies [11]. Our juvenile SHRs cohort, though not tested for blood acetate levels, displayed an increase in intestinal acetate-producing bacteria. From these observations, we hypothesize that the diminished serum acetate in adult SHRs arises from hypertension-induced shifts in the intestinal microenvironment. Such changes may affect the bacterial composition and lead to a reduction in acetate-producing bacteria, consequently lowering acetate levels. Yet, these hypotheses warrant further investigation for confirmation.

A growing compilation of findings from various clinical studies increasingly supports the hypothesis that disturbances in gut microecology could influence the risk and progression of a range of neurological disorders, such as Alzheimer’s disease, Parkinson’s disease, multiple sclerosis, and autism spectrum disorders [45]. The communication between gut microbes and the brain occurs through numerous ways, including the autonomic and enteric nervous systems, the immune response, and the release of metabolites like SCFAs. While earlier animal studies have indicated that SCFAs, including acetate, propionate and butyrate, can lower blood pressure in models of hypertension [27, 46, 47], the focus has primarily been on how they affect the peripheral mechanisms, leaving the central mechanisms largely unexplored. In our current research, we have uncovered an innovative mechanism by which the SCFAs acetate exerts antihypertensive effects. This mechanism involves the regulation of microglia and astrocytes to dampen neuroinflammation, which in turn diminishes sympathetic nervous system activity and lowers blood pressure. Recent research evidence demonstrated that microglia possess receptors for SCFAs (GPR41), suggesting a potential pathway for their regulatory influence [48]. Overall, our findings shed new light on the intricate interplay between the gut microbiome and the nervous system.

In summary, our research indicates that hypertension may induce or intensify intestinal dysbiosis, leading to changes in the concentrations of SCFAs and acetate. Acetate has been observed to influence the morphology and functionality of microglia and astrocytes, reduce neuroinflammation and sympathetic efferent activity, and promote hypotensive responses. Subsequent studies are encouraged to delve deeper into the processes through which acetate affects microglial and astrocytic regulation.

### Electronic supplementary material

Below is the link to the electronic supplementary material.

Supplementary Material 1

Supplementary Material 2

Supplementary Material 3

Supplementary Material 4

Supplementary Material 5

Supplementary Material 6

Supplementary Material 7

Supplementary Material 8

## Data Availability

All data are available upon reasonable request.
