# Supplementary figures and images for: Microbiota-derived acetate attenuates neuroinflammation in rostral ventrolateral medulla of spontaneously hypertensive rats
Source: J Neuroinflammation. 2024 Apr 18;21:101. doi: 10.1186/s12974-024-03061-3 (PMC11025215; doi:10.1186/s12974-024-03061-3)

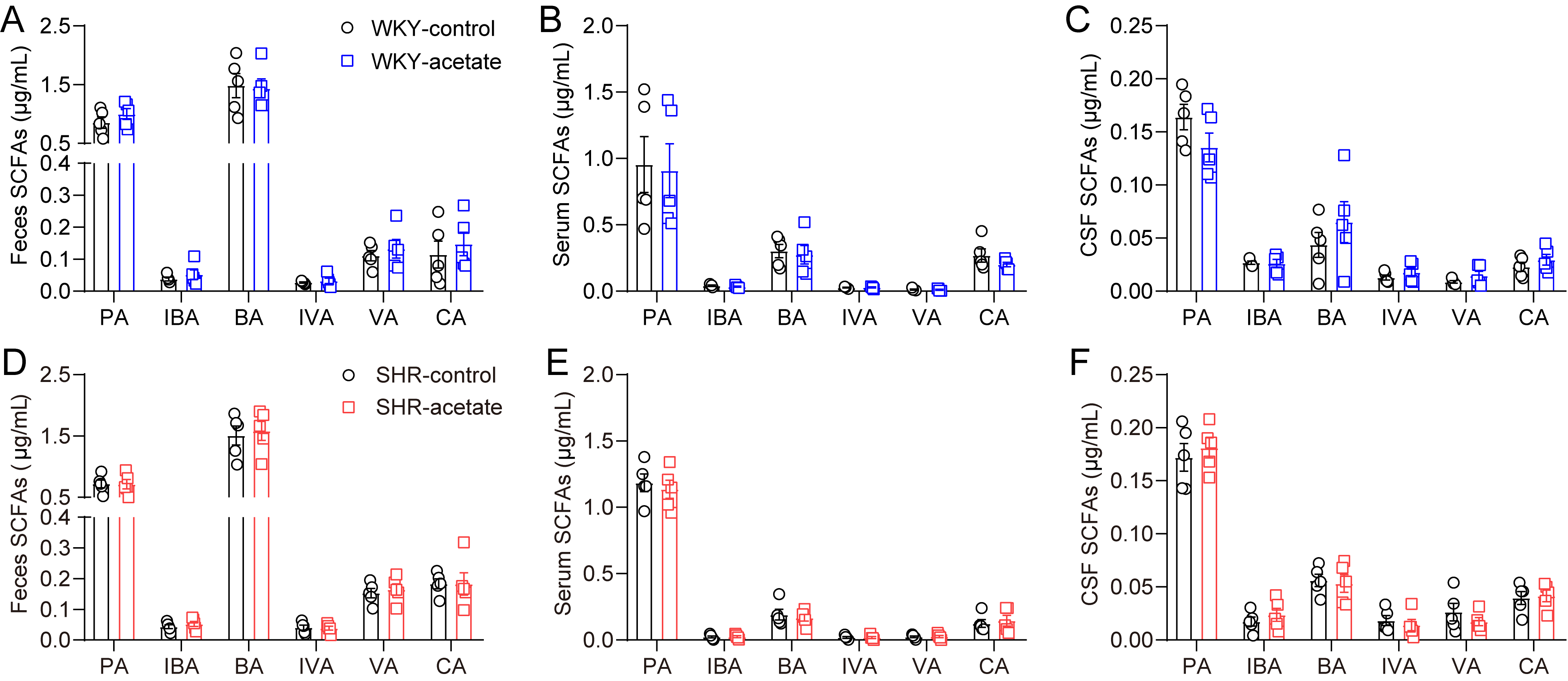

Supplement: Supplementary file 1 — Supplementary Material 1 [file 12974_2024_3061_MOESM1_ESM.tif]

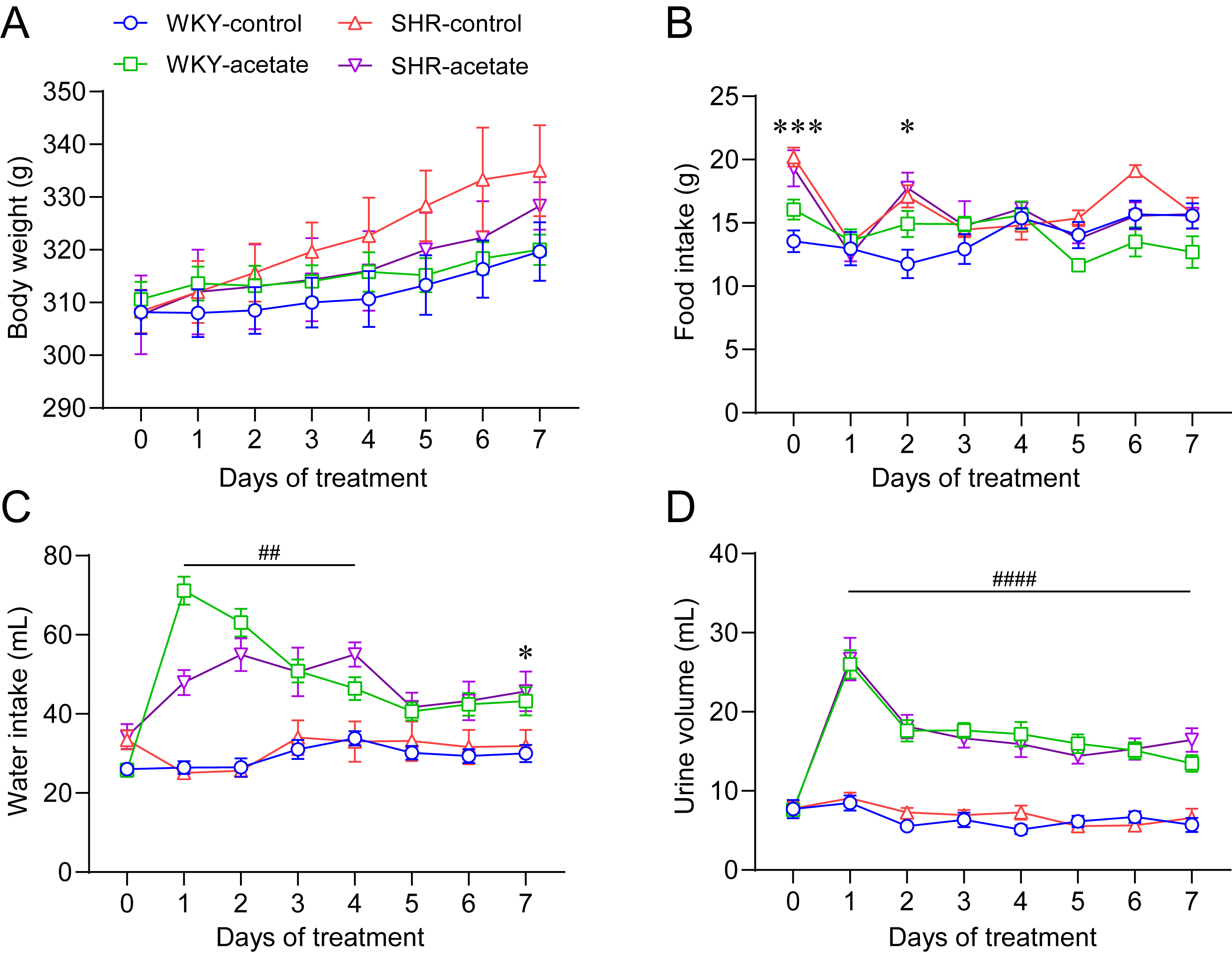

Supplement: Supplementary file 2 — Supplementary Material 2 [file 12974_2024_3061_MOESM2_ESM.tif]

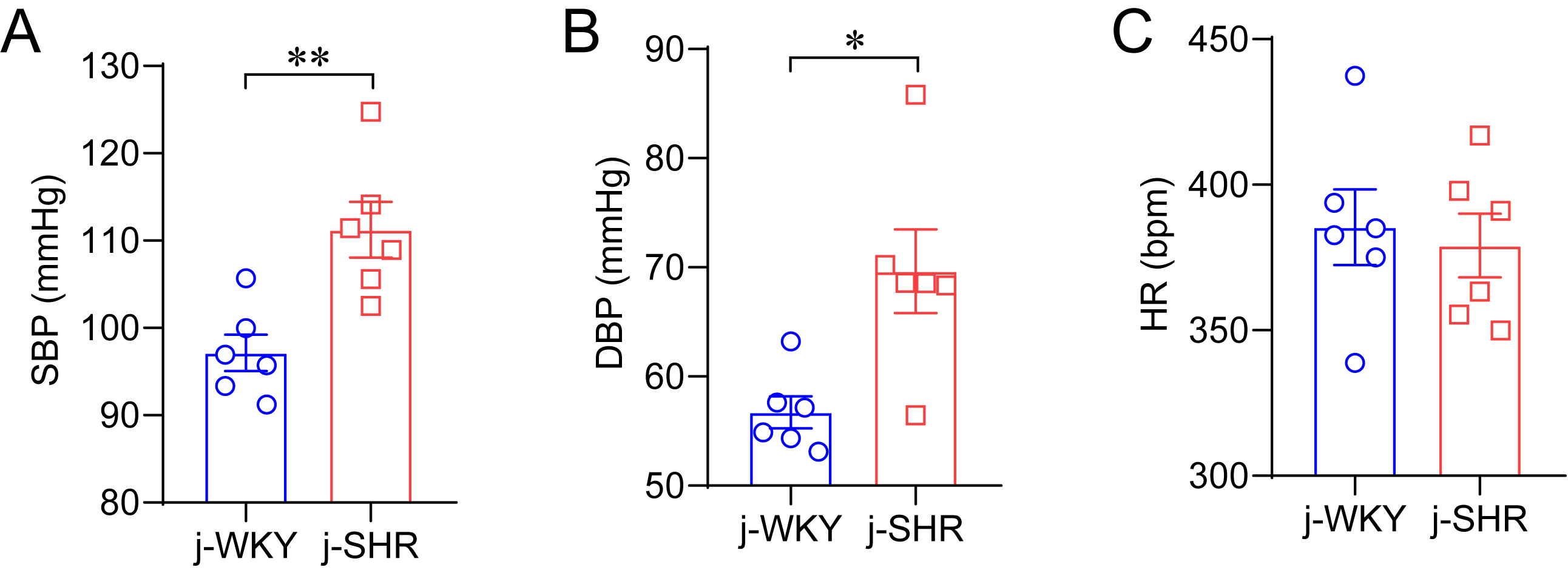

Supplement: Supplementary file 3 — Supplementary Material 3 [file 12974_2024_3061_MOESM3_ESM.tif]

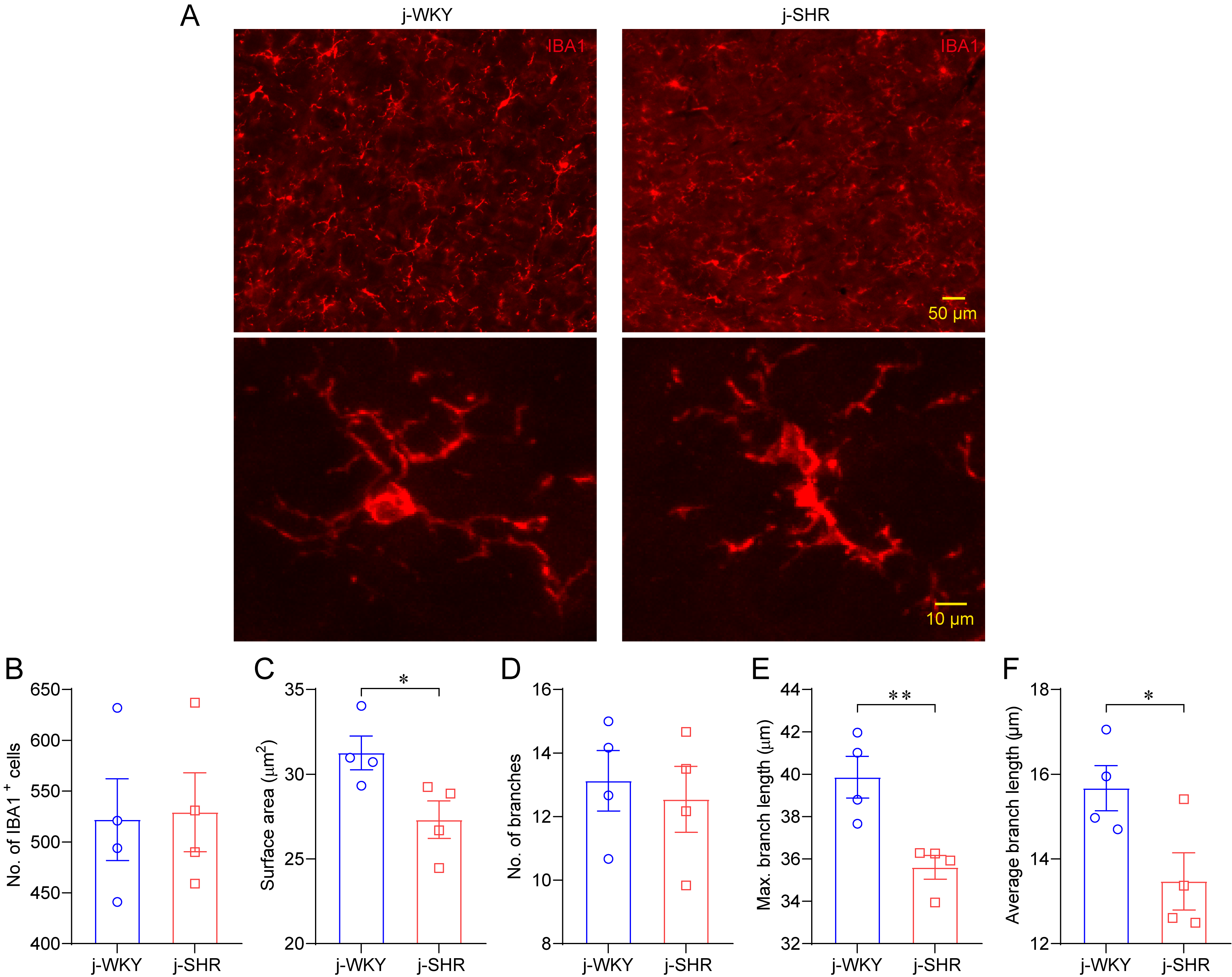

Supplement: Supplementary file 4 — Supplementary Material 4 [file 12974_2024_3061_MOESM4_ESM.tif]

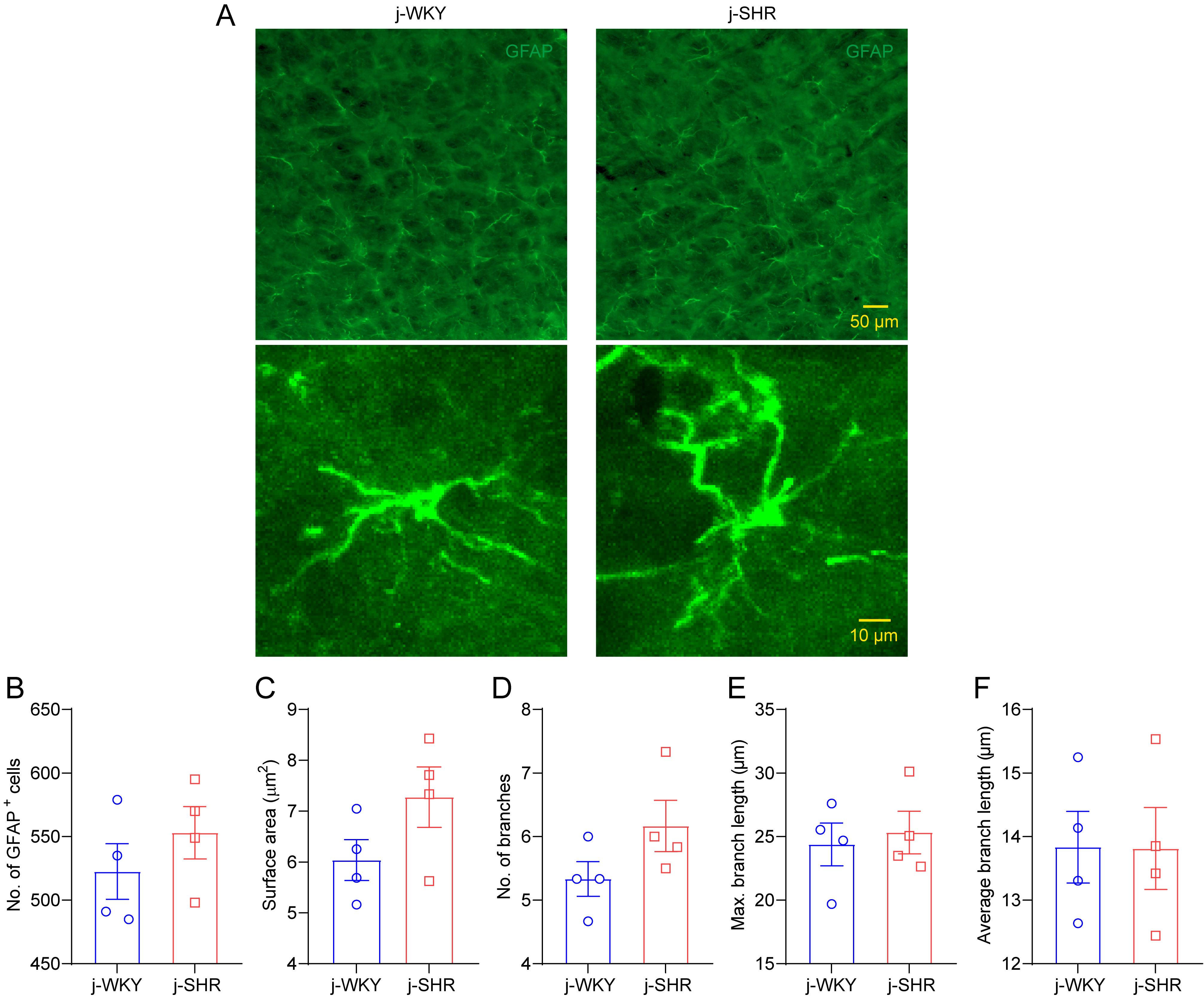

Supplement: Supplementary file 5 — Supplementary Material 5 [file 12974_2024_3061_MOESM5_ESM.tif]

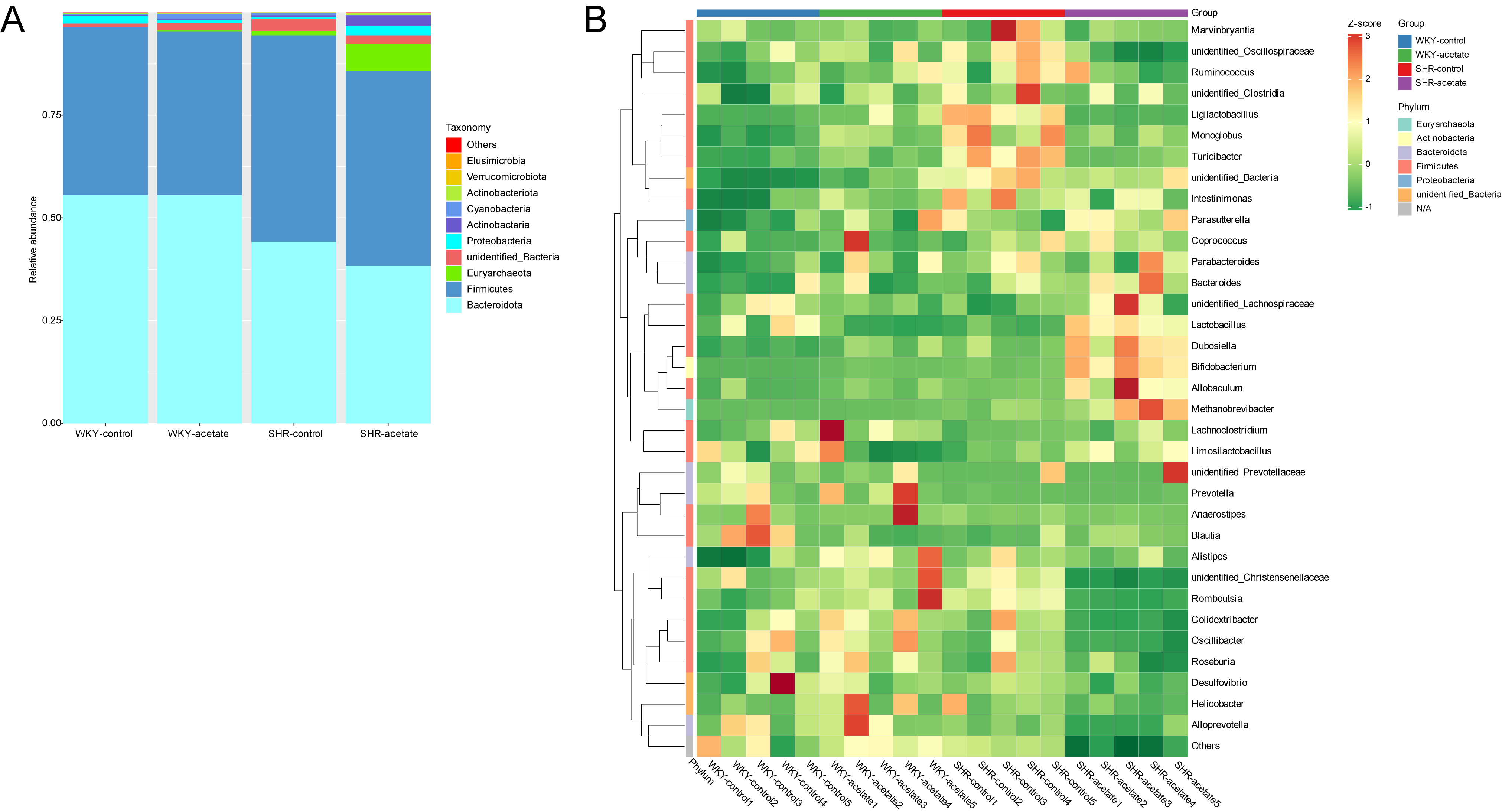

Supplement: Supplementary file 6 — Supplementary Material 6 [file 12974_2024_3061_MOESM6_ESM.tif]

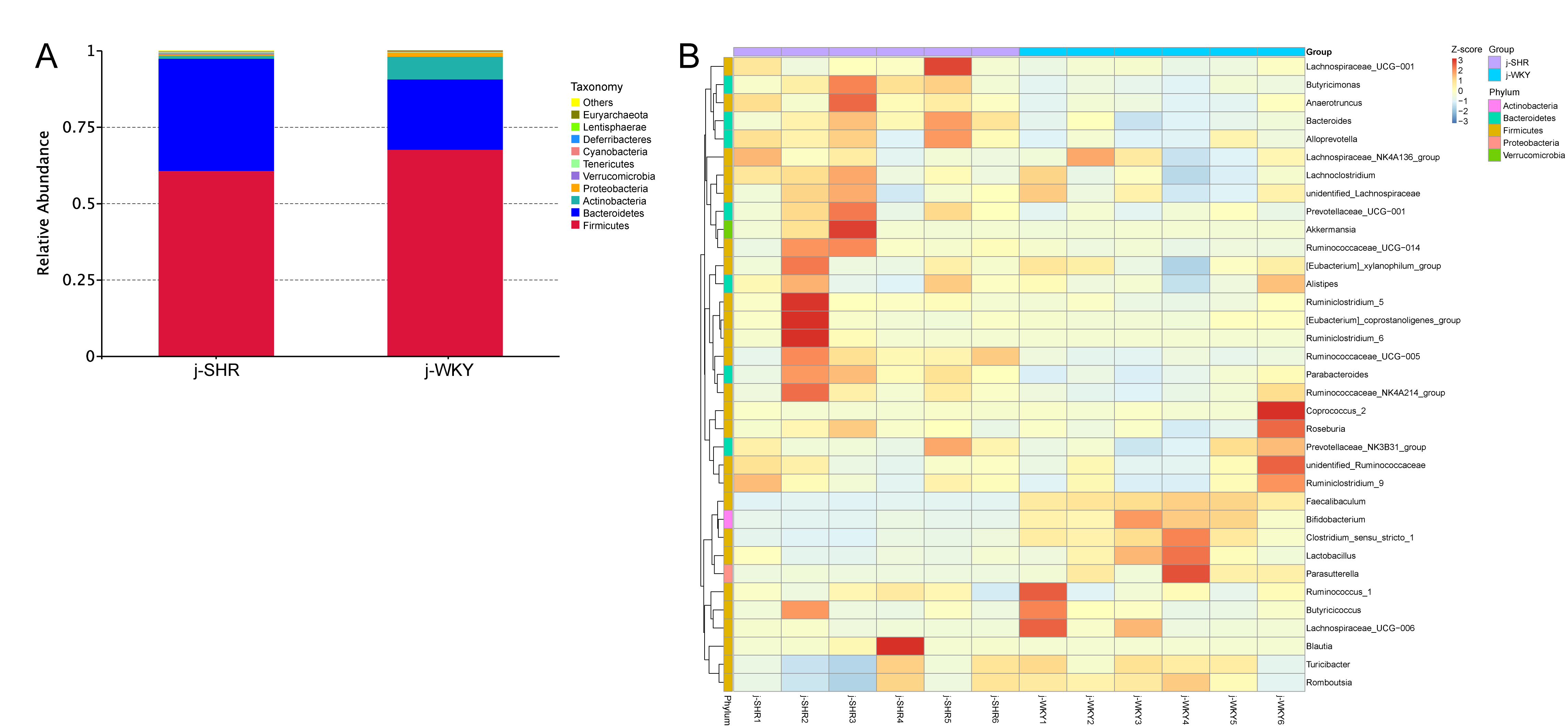

Supplement: Supplementary file 7 — Supplementary Material 7 [file 12974_2024_3061_MOESM7_ESM.tif]
