# Supplementary material for: Microbiota-derived acetate attenuates neuroinflammation in rostral ventrolateral medulla of spontaneously hypertensive rats
Source: J Neuroinflammation. 2024 Apr 18;21:101. doi: 10.1186/s12974-024-03061-3 (PMC11025215; doi:10.1186/s12974-024-03061-3)
Supplement: Supplementary file 8 — Supplementary Material 8 [file 12974_2024_3061_MOESM8_ESM.docx]

**Microbiota-derived acetate attenuates** **neuroinflammation in rostral ventrolateral medulla of spontaneously hypertensive rats**

Xiaopeng Yin^1*^, Changhao Duan^1*^, Lin Zhang^1*^, Yufang Zhu^1^, Yueyao Qiu^1^, Kaiyi Shi^1^, Sen Wang^2^, Xiaoguang Zhang^3^, Huaxing Zhang^3^, Yinchao Hao^1^, Fang Yuan^1,4*^, Yanming Tian^1,4*^

**Figure S1. Effect of acetate supplementation on SCFAs in WKY rats and SHRs**

The concentrations of SCFAs were measured in feces, serum and cerebrospinal fluid of WKY rats (A-C) and SHRs (D-F) after acetate supplementation. n = 5 for each group.

**Figure S2. Effects of acetate supplementation on general metabolic parameters in rats**

Effect of acetate supplementation on body weight (A), food intake (B), water intake (C) and urine output (D) in rats. n = 3 - 6. * *P* < 0.05, *** *P* < 0.001 SHR-control *vs*. WKY-control. **^##^** *P* < 0.01, **^####^** *P* < 0.0001 acetate treatment group *vs*. corresponding control group.

**Figure S3. Cardiovascular parameters in prehypertensive juvenile SHRs (j-SHRs)**

The SBP (A), DBP (B) and HR (C) of 5-week-old SHRs (j-SHRs) and age-matched WKY rats (j-WKYs). n = 6 for each group. * *P* < 0.05, ** *P* < 0.01.

**Figure S4. Morphologic characteristics of microglia in the RVLM of juvenile SHRs**

A, representative confocal photomicrographs of IBA^+^ microglia in RVLM in 5-week-old juvenile SHRs and age-matched WKY rats. Quantitative analysis of the cell density (B), soma size (C), branches number (D), the longest branches (E) and average lengths of the branches (F) of the IBA^+^ microglia. n = 4 for each group. * *P* < 0.05, ** *P* < 0.01.

**Figure S5. Morphologic manifestations of astrocytes in the RVLM of juvenile SHRs**

A, representative confocal photographs of GFAP^+^ astrocytes in RVLM in 5-week-old juvenile SHRs and age-matched WKY rats. Quantitative analysis of the cell density (B), soma size (C), branches number (D), the longest branches (E) and average lengths of the branches (F) of the GFAP^+^ astrocytes. n = 4 for each group.

**Figure S6. Relative abundance of gut microbes in SHRs**

A, differences in intestinal flora at the top10 phylum level between SHRs and WKY rats. B, differences in intestinal flora at the top 35 genus level between SHRs and WKY rats. n = 5 for each group.

**Figure S7. Relative abundance of gut microbes in 5-week-old juvenile SHRs**

A, differences in gut microbes at the top10 phylum level between j-SHRs and age-matched j-WKYs. B, differences in gut microbes at the top 35 genus level between j-SHRs and j-WKYs. n = 6 for each group.
